# Common variants in *LTBP*3 gene contributed to the risk of hip osteoarthritis in Han Chinese population

**DOI:** 10.1042/BSR20192999

**Published:** 2020-06-09

**Authors:** Tianyun Zhao, Junji Zhao, Chi Ma, Jie Wei, Bo Wei, Jibin Liu

**Affiliations:** 1 Department of Orthopedics, the First Hospital of Tianshui City, Tianshui, China; 2 Department of Sports Medicine, the First Hospital of Tianshui City, Tianshui, China; 3 Department of Oncology Research, the Affiliated Oncology Hospital of Nantong University, Nantong, China

**Keywords:** genetic association, hip osteoarthritis, LTBP3 gene, single nucleotide polymorphism

## Abstract

Osteoarthritis (OA) is a common chronic joint disease affected by environmental and genetic factors. The *LTBP3* gene may be involved in the occurrence and development of OA by regulating TGF-β activity and the TGF-β signaling pathway. A total of 2780 study subjects, including 884 hip OA cases and 1896 controls, were recruited. Nine tag single-nucleotide polymorphisms (SNPs) located within the *LTBP3* gene region were selected for genotyping. Genetic association analyses were performed at both the genotypic and allelic levels. GTEx data were extracted to investigate the functional consequence of significant SNPs. SNP rs10896015 was significantly associated with the risk of hip OA at both the genotypic (*P*=0.0019) and allelic levels (*P*=0.0009). The *A* allele of this SNP was significantly associated with a decreased risk of HOA (OR [95%CI] = 0.79 [0.69–0.91]). This SNP was also significantly associated with the clinical severity of hip OA. SNP rs10896015 could affect the gene expression of 11 genes, including *LTBP3*, in multiple human tissues based on GTEx data. We obtained evidence for a genetic association between the *LTBP3* gene and hip OA susceptibility and clinical severity based on Chinese Han populations. Our findings replicated the association signals reported by a recent genome-wide association study and deepen the basic understanding of osteoarthritis pathology.

## Introduction

Osteoarthritis (OA) is a common chronic joint disease characterized by primary or secondary degeneration of articular cartilage and bone hyperplasia [1]. Of these, articular cartilage degeneration is the earliest and most important pathological change [2]. The most common pathological changes of OA occur in the knee joint and hip joint. Although the prevalence of hip OA (HOA) is lower than knee OA, HOA is more likely to cause disability and death in the elderly [3]. The lifetime risk of developing HOA is approximately 25%, which leads to a socioeconomic and healthcare burden [4]. Similar to other chronic and complex diseases, HOA is also affected by environmental and genetic factors. Age and gender can influence the incidence of HOA, and multiple genetic studies have confirmed the importance of genetic factors [5]. Twin and family studies indicated that the heritability for HOA is approximately 60% [6]. Hence, it is necessary to identify the genes responsible for susceptibility to HOA through candidate gene studies.

Recently, single-nucleotide polymorphism (SNP) rs10896015 in the latent TGF-β binding protein3 (*LTBP3*) gene was significantly associated with HOA in the British population through a genome-wide association study (GWAS) that included 77,052 OA patients and 378,169 controls [7]. To the best of our knowledge, this is the first association study between the HOA and *LTBP3* genes. LTBP3 is a member of the latent TGF-β binding protein, which has four isoforms (LTBP1–4) in the human genome [8]. The cysteine-rich domain of LTBP-1, 3, and 4 can covalently bind latent TGF-β [9]. Through the use of human osteoarthritic osteoblasts, researchers have found increased levels of TGF-β, which may up-regulate cartilage matrix-degrading enzyme expression and ultimately result in OA [10,11]. In addition, researchers have also found increased expression levels of TGF-β in articular cartilage and osteophytes in an OA mouse model, and this phenomenon can be reversed by endogenous TGF-β [12]. These results suggested that expression changes in TGF-β could affect OA directly. A recent study demonstrated that up-regulated LTBP1 caused activation of the TGF-β signaling pathway in human OA fibroblast-like synoviocytes, which may lead to the aggravation of OA [13]. Hence, the *LTBP3* gene may also be involved in the occurrence and development of OA by regulating TGF–β activity and the TGF-β signaling pathway. However, few studies have focused on the relationship between the *LTBP3* gene and HOA, and the only association study was conducted in individuals of European ancestry. To confirm whether the common variants in the *LTBP3* gene were universally associated with the risk of HOA, we systematically investigated the association of the *LTPB3* gene with the risk of HOA in the Han Chinese population in the present study.

## Methods

### Study subjects

A total of 2780 study subjects, including 884 HOA cases and 1896 controls, were recruited from the Second Hospital of Xi'an Jiaotong University from March 2014 to February 2019. HOA patients were diagnosed by three independent physicians based on criteria published by the American College of Rheumatology. Demographic information, including age, gender, BMI, smoking and alcohol consumption, was collected through a questionnaire. HOA patients with Kellgren–Lawrence (KL) grading scores of 2 or more were included. Study subjects with no symptoms and family history of arthritis or any other joint-related disorders were enrolled as controls. Study subjects were excluded if they had (1) inflammatory arthritis, (2) post-traumatic or post-septic arthritis, (3) skeletal or developmental dysplasia, or (4) systemic or organic diseases. The present study was approved by the institutional review board of the Second Hospital of Xi’an Jiaotong University. Informed consent forms were signed and obtained from all study subjects.

### SNP selection and genotyping

For SNP selection, 1000 Genomes Chinese Han Beijing (CHB) data were used as reference data. We extracted all SNPs with MAFs greater than 0.03 within the *LTBP3* gene region in 1000 Genome CHB data, resulting in a SNP set of 19. Then, tag SNPs (*r*^2^≥0.8) were identified within this SNP set based on the method proposed by Gabriel et al*.* [14]. Finally, a total of 9 tag SNPs located within the *LTBP3* gene region were selected for genotyping (Supplementary Table S1). We extracted genomic DNA from peripheral blood leukocytes according to the manufacturer’s protocol (Genomic DNA Kit, Axygen Scientific, Inc., CA, U.S.A.). A high-throughput Sequenom MassARRAY platform (Sequenom, San Diego, CA, U.S.A.) was utilized for SNP genotyping. Briefly, the signals from the platform were automatically analyzed using Sequenom Typer 4.0 software, and genotype data were generated from the processed results [15]. To estimate the genotyping quality, 5% of random samples were repeated for genotyping. With a concordance rate of 100%, the quality of genotyping data was confirmed. The case/control status of the samples was blinded to the technicians during the genotyping process [16].

### Statistical analyses

We have conducted a comprehensive power statistical analysis for the present study using GAS power calculator (http://csg.sph.umich.edu/abecasis/cats/gas_power_calculator/). The results indicated that our sample size could achieve approximately 71% statistical power for genetic association (Supplementary Table S2 and Supplementary Figure S1). Single marker-based association analyses were conducted at both allelic and genotypic levels. χ^2^ tests were performed for both levels of analyses. Further association analysis was conducted between significant SNPs and KL grading scores in the HOA patient group. Genetic association analyses were performed by Plink [17]. Linkage disequilibrium structures of our selected SNPs were visualized using Haploview [18]. Bonferroni corrections were applied to address multiple comparisons. In general, the threshold of the *P*-value for single marker-based association analyses was 0.05/9≈0.006. To further investigate the functional consequences of the significant hits identified from genetic association analyses, we extracted the expression quantitative trait loci (eQTL) data obtained from multiple human tissues from the GTEx database [19].

## Results

**Figure 1:**
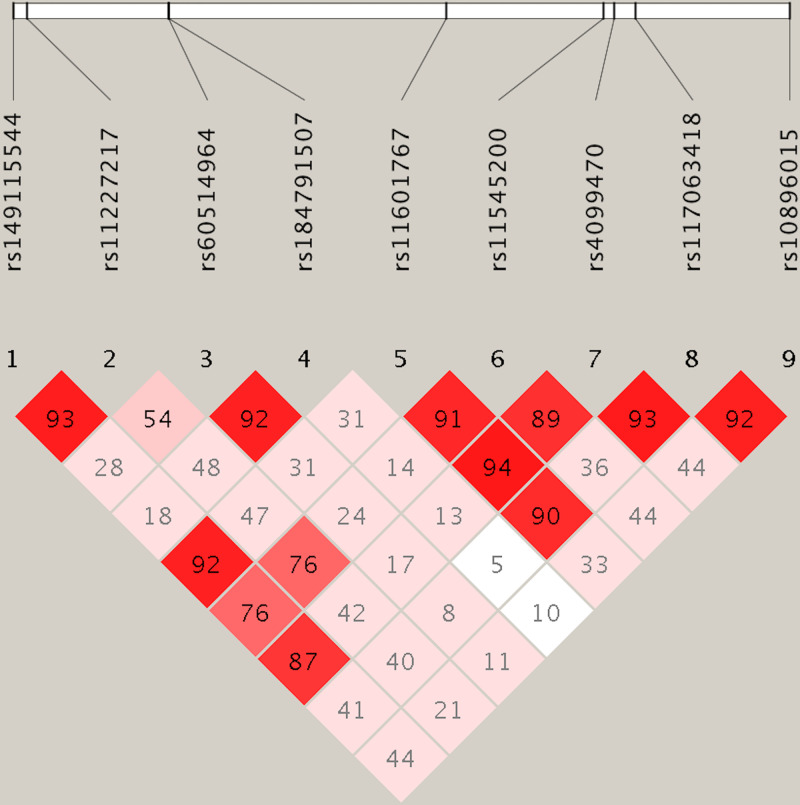
Linkage disequilibrium plot of the selected SNPs Values of D’ are indicated in each cell.

**Figure 2:**
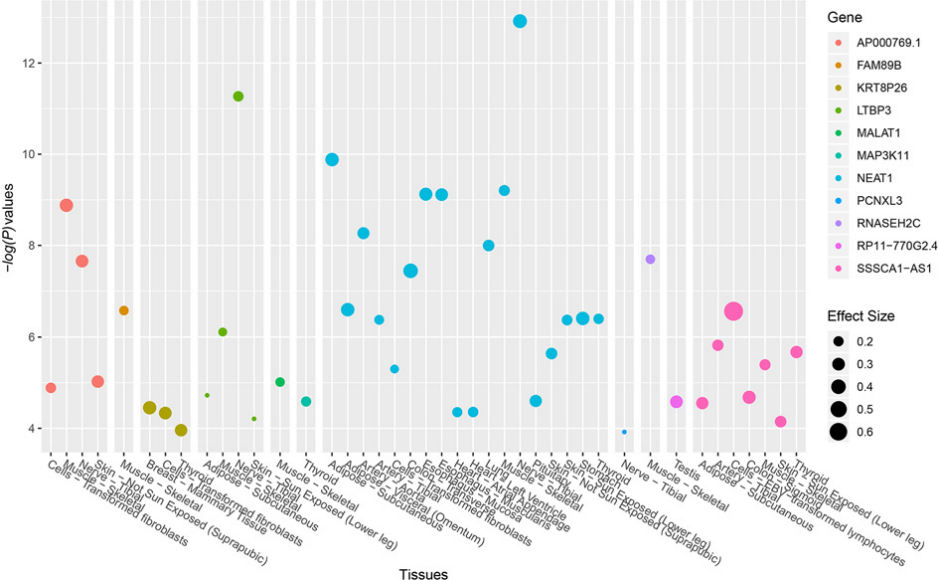
eQTL signals of SNP rs10896015 achieved genome-wide significance Data were extracted from the GTEx database.

A total of 884 HOA cases and 1896 healthy controls were recruited in the present study (Table 1). No significant differences were identified between HOA patients and healthy controls in multiple demographic and clinical variables, including age, gender, BMI, smoking and alcohol consumption status. In the 884 HOA patients, there were 372 with KL scores of 2 (42%), 325 with KL scores of 3 (37%) and 187 with KL scores of 4 (21%). SNP rs10896015, an intronic SNP located in gene *LTBP3*, was significantly associated with the disease status of HOA at both the genotypic (*P*=0.0019) and allelic levels (*P*=0.0009). The *A* allele of this SNP, which was the minor allele, was significantly associated with a decreased risk of HOA (OR [95%CI] = 0.79 [0.69–0.91], Table 2). We further examined the association between SNP rs10896015 and KL grading scores in the HOA patients. We identified that this SNP was significantly associated with KL grading score (Table 3). There were significantly more patients with GG genotypes in the KL-4 group than in the KL-2 and KL-3 groups (69% vs. 62% vs. 62%, respectively). The LD structure of the nine selected SNPs is shown in Figure 1. We explored SNP rs10896015 in the GTEx database and identified that the genotypes of this SNP were significantly associated with the gene expression of *LTBP3* in various human tissues (Supplementary Table S3 and Figure 2). Interestingly, in addition to *LTBP3*, SNP rs10896015 was also significantly associated with the gene expression levels of several other genes, including *AP000769.1, FAM89B, KRT8P26, MALAT1, MAP3K11, NEAT1, PCNXL3, RNASEH2C, RP11-770G2.4*, and *SSSCA1-AS1*.

**Table 1:** Demographic information of the study subjects

Variables	Cases (*N*=884)	Controls (*N*=1896)	Statistics	*P*-value
Age, years	61.8 ± 7.9	61.7 ± 8.2	*T* = 0.35	0.73
BMI, kg/m^2^	25.9 ± 1.4	25.8 ± 1.6	*T* = 1.56	0.12
Gender (%)				
*Male*	387 (44)	826 (44)		
*Female*	497 (56)	1070 (56)	χ^2^ = 0.0041	0.95
Smoking (%)				
*Yes*	262 (30)	559 (29)		
*No*	622 (70)	1337 (71)	χ^2^ = 0.0015	0.97
Alcohol consumption (%)				
*Yes*	229 (26)	488 (26)		
*No*	655 (74)	1408 (74)	χ^2^ = 0.0022	0.96
KL grade (%)				
*KL-2*	372 (42)			
*KL-3*	325 (37)			
*KL-4*	187 (21)			

**Table 2:** Results of single marker-based association analyses

SNP	Position	Status	Genotypes			*χ^2^	*P*-value	Alleles		χ^2^	*P*-value	OR [95%CI]
			GG	GA	AA			G	A			
rs149115544	65539637	Cases	6	104	774			116	1652			
		Controls	9	220	1667	0.49	0.78	238	3554	0.16	0.69	1.05[0.83–1.32]

SNP	Position	Status	Genotypes			*χ^2^	*P*-value	Alleles		χ^2^	*P*-value	OR [95%CI]
			TT	TC	CC			T	C			
rs11227217	65539931	Cases	27	267	590			321	1447			
		Controls	65	576	1255	0.29	0.87	706	3086	0.17	0.68	0.97[084–1.12]

SNP	Position	Status	Genotypes			*χ^2^	*P*-value	Alleles		χ^2^	*P*-value	OR [95%CI]
			GG	GA	AA			G	A			
rs60514964	65542976	Cases	9	129	746			147	1621			
		Controls	15	262	1619	0.69	0.71	292	3500	0.63	0.43	1.09[0.88–1.34]

SNP	Position	Status	Genotypes			*χ^2^	*P*-value	Alleles		χ^2^	*P*-value	OR [95%CI]
			AA	AG	GG			A	G			
rs184791507	65542980	Cases	4	78	802			86	1682			
		Controls	6	163	1727	–	0.80	175	3617	0.17	0.68	1.06[0.81–1.38]

SNP	Position	Status	Genotypes			*χ^2^	*P*-value	Alleles		χ^2^	*P*-value	OR [95%CI]
			CC	CT	TT			C	T			
rs11601767	65548911	Cases	115	407	362			637	1131			
		Controls	254	884	785	0.25	0.88	1392	2400	0.24	0.62	0.97[0.86–1.09]

SNP	Position	Status	Genotypes			*χ^2^	*P*-value	Alleles		χ^2^	*P*-value	OR [95%CI]
			AA	AG	GG			A	G			
rs11545200	65552280	Cases	5	95	784			105	1663			
		Controls	6	201	1689	0.97	0.62	213	3579	0.23	0.63	1.06[0.83–1.35]

SNP	Position	Status	Genotypes			*χ^2^	*P*-value	Alleles		χ^2^	*P*-value	OR [95%CI]
			TT	TC	CC			T	C			
rs4099470	65552515	Cases	11	196	677			218	1550			
		Controls	28	425	1443	0.27	0.88	481	3311	0.14	0.71	0.97[0.82–1.15]

SNP	Position	Status	Genotypes			*χ^2^	*P*-value	Alleles		χ^2^	*P*-value	OR [95%CI]
			AA	AG	GG			A	G			
rs117063418	65552969	Cases	5	94	785			104	1664			
		Controls	8	197	1,691	0.31	0.86	213	3579	0.16	0.69	1.05[0.82–1.34]

SNP	Position	Status	Genotypes			*χ^2^	*P*-value	Alleles		χ^2^	*P*-value	OR [95%CI]
			**AA**	**AG**	**GG**			**A**	**G**			
**rs10896015**	**65556254**	**Cases**	**30**	**292**	**562**			**352**	**1416**			
		**Controls**	**116**	**675**	**1105**	**12.48**	**0.0019**	**907**	**2885**	**11.07**	**0.0009**	**0.79[0.69–0.91]**

The threshold of the *P*-value is 0.05/9≈0.006. Significant markers are highlighted in bold.

* Fisher’s exact test was applied for sparse contingency tables.

**Table 3:** Results of association between KL grading scores and genotypes of SNP rs10896015

KL grade	Genotypes			χ^2^	*P*-value
	AA	AG	GG		
KL-2 (%)	9 (3)	130 (35)	233 (62)		
KL-3 (%)	7 (2)	118 (36)	200 (62)		
KL-4 (%)	14 (7)	44 (24)	129 (69)	19.45	0.0006

## Discussion

In the present study, we found that an intronic SNP of the *LTBP3 gene*, SNP rs10896015, was significantly associated with HOA disease status based on study subjects with Chinese Han ancestry. To the best of our knowledge, our study was the first to link *LTBP3* to HOA in a Chinese Han population. SNP rs10896015 was significantly associated with HOA in a recently published GWAS using UK Biobank data, which are mainly based on European populations [7]. The effect direction of SNP rs10896015 was the same in our study and the recent GWAS. Thus, our study could be considered as a validation for this recent GWAS. In addition, in the present study, we took it one step further to show that SNP rs10896015 was not only associated with the disease status of HOA but also significantly associated with the clinical severity of HOA.

The product of *LTBP3* is a member of latent TGF-β binding proteins. A large number of studies have indicated that TGF-β plays an important role in the formation of articular chondrocytes and the homeostasis of cartilage matrix [20,21]. Cardiovascular defects were found in an LTBP3 knockdown zebrafish model, which had been rescued by a constitutively active TGFβ type 1 receptor [22]. This result suggests that LTBP3 may also be a regulator of TGF-β. Moreover, LTBP3 (LTBP3-/-) knockout mice showed the same pathological changes in joints as OA, such as osteophytes, ossified and fibrotic areas on the articular surface and the absence of articular cartilage [23]. The same result was consistent with the perturbed TGF-β signaling pathway in mouse long bones, which further strengthens the evidence linking LTBP3 and TGF-β [24].

With the fast development of target sequencing, numerous susceptibility variants of complex diseases have been identified, such as schizophrenia [25–27] (Zhang and others 2018; Han and others 2019; Guan and others 2020a). Given that it is difficult to draw reliable conclusions only from single SNP association analyses [28–32] (Zhu and others 2016; Sun and others 2017; Zhang and others 2017; Li and others 2018; Guan and others 2020b), although SNP rs10896015 is an intronic SNP that cannot alter the structure of the protein encoded by *LTBP3*, our analyses using GTEx data confirmed that it was significantly associated with the gene expression of *LTBP3* in multiple human tissues. Therefore, SNP rs10896015 might be a DNA variant with functional significance but not just a surrogate of some underlying DNA variants that were not genotyped in this present study. In addition, the eQTL analysis using GTEx data also provides interesting results. It seemed that SNP rs10896015 was an eQTL for *LTBP3* and for several other genes located near SNP rs10896015 and the *LTBP3* gene. Of these, some genes, such as *AP000769.1* and *RP11-770G2.4*, had unknown functions (most likely some nonfunctional peptides) and were not worthy of discussion. However, a long noncoding gene, *NEAT1*, was of particular interest. First, SNP rs10896015 was a significant eQTL signal in a large number of human tissues for *NEAT1*. Second, *NEAT1* was linked to osteogenic differentiation in human bone marrow-derived mesenchymal stem cells in a recent study and therefore might play a role in the metabolism of bone tissues [33]. In this sense, we have now identified a significant SNP for HOA; however, the genetic location of this SNP was unclear. One strategy was to physically map this SNP to the *LTBP3* gene because this SNP is located within its gene region. This is a common SNP-gene mapping strategy used in most (if not all) GWAS. On the other hand, this SNP could probably be functionally mapped to other genes, such as *NEAT1*, when eQTL data were integrated and considered. In the present study, our data could not provide enough information to address this issue. In the future, experimental research based on animal models will be conducted to clarify this point.

Our study has from several limitations. First, as a genetic association study, the association signals identified in our study might be confounded by population stratification. Unfortunately, as a candidate gene-based association study, we could use common methods utilized in GWAS, such as principal component analysis, to control this most commonly identified confounder in population genetics studies. Another limitation is that the information coverage of our selected SNPs might be insufficient to explore the whole functional regions of *LTBP3*. Only 9 tag SNPs within the gene region were genotyped in this present study, and SNPs located at ±20 kb up/downstream of *LTBP3*, which are important functional regions for the regulation of gene expression, were not investigated in this study. This strategy of SNP selection enabled us to maximize the information coverage under limited experimental expense, although it might miss significant functional regions of the targeted gene.

In summary, in the present study, we obtained evidence for a genetic association between *LTBP3* and HOA disease status and clinical severity based on Chinese Han populations. Our findings replicated the association signals reported by recent GWAS and could deepen the basic understanding of osteoarthritis pathology.

## Supplementary Material

Supplementary Figure S1 and Tables S1-S3

## Abbreviations

eQTL: expression quantitative trait loci
HOA: hip OA
KL: Kellgren–Lawrence
OA: osteoarthritis
SNP: single-nucleotide polymorphism
