# Supplementary material for: Common variants in LTBP3 gene contributed to the risk of hip osteoarthritis in Han Chinese population
Source: Biosci Rep. 2020 Jun 9;40(6):BSR20192999. doi: 10.1042/BSR20192999 (PMC7284319; doi:10.1042/BSR20192999)
Supplement: Supplementary Figure S1 and Tables S1-S3 [file BSR-2019-2999_supp.pdf]

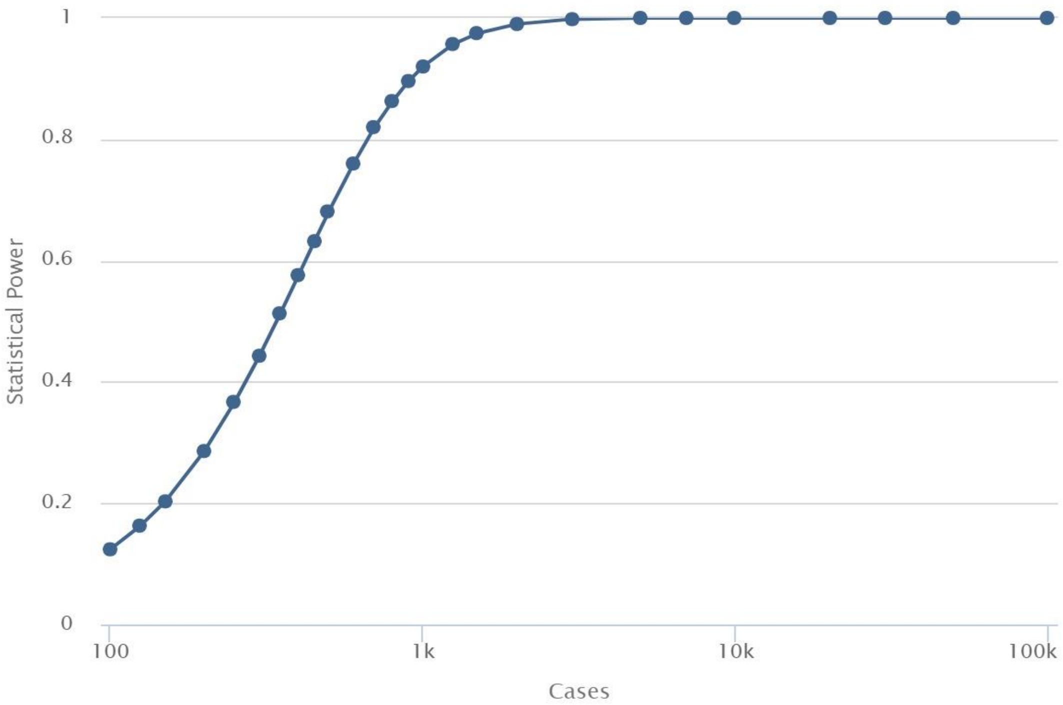

Supplemental Figure S1. Results of the statistical analysis for the present study. Expected number of cases versus statistical power of the present study.

Supplemental Table S1. Basic information of the 9 selected SNPs for genotyping

CHR	POS	SNP	Minor Allele	Major Allele	MAF	HWE	Function
11	65539637	rs149115544	G	A	0.06	0.55	intron
11	65539931	rs11227217	T	C	0.18	1.00	intron
11	65542976	rs60514964	G	A	0.08	0.25	intron
11	65542980	rs184791507	A	G	0.05	0.29	intron
11	65548911	rs11601767	C	T	0.36	0.92	intron
11	65552280	rs11545200	A	G	0.06	1.00	missense
11	65552515	rs4099470	T	C	0.13	0.68	intron
11	65552969	rs117063418	A	G	0.06	0.38	coding-synon
11	65556254	rs10896015	A	G	0.23	0.34	intron

CHR: chromosome; POS: position; MAF: minor allele frequency; HWE: *P*-value of Hardy-Weinberg equilibrium tests in controls.

Supplemental Table S2. Parameters utilized in statistical power analysis

Parameters	Values	Note
Mode	Additive	-
Relative risk	1.3	-
MAF	0.13	Average MAF of the 9 SNPs in 1000 genome CHB data
Cases: Controls	0.464	-
α	0.006	0.05/9
Prevalence	4.2%	Ref: PMID 25775186

Supplemental Table S3. eQTL signals achieved genome-wide significance for SNP rs10896015 in multiple human tissues based on data extracted from GTEx database

GENE	SNP	Ref_Alt	P-Value	NES	Tissue
AP000769.1	rs10896015	G_A	1.30×10^{-5}	0.21	Cells - Transformed fibroblasts
AP000769.1	rs10896015	G_A	1.30×10^{-9}	0.33	Muscle - Skeletal
AP000769.1	rs10896015	G_A	2.20×10^{-8}	0.3	Nerve - Tibial
AP000769.1	rs10896015	G_A	9.40×10^{-6}	0.29	Skin - Not Sun Exposed (Suprapubic)
FAM89B	rs10896015	G_A	2.60×10^{-7}	0.18	Muscle - Skeletal
KRT8P26	rs10896015	G_A	3.50×10^{-5}	-0.33	Breast - Mammary Tissue
KRT8P26	rs10896015	G_A	4.60×10^{-5}	-0.3	Cells - Transformed fibroblasts
KRT8P26	rs10896015	G_A	1.10×10^{-4}	-0.29	Thyroid
LTBP3	rs10896015	G_A	1.90×10^{-5}	0.11	Adipose - Subcutaneous
LTBP3	rs10896015	G_A	7.70×10^{-7}	0.16	Muscle - Skeletal
LTBP3	rs10896015	G_A	5.40×10^{-12}	0.21	Nerve - Tibial
LTBP3	rs10896015	G_A	6.20×10^{-5}	0.11	Skin - Sun Exposed (Lower leg)
MALAT1	rs10896015	G_A	9.60×10^{-6}	-0.18	Muscle - Skeletal
MAP3K11	rs10896015	G_A	2.60×10^{-5}	-0.2	Thyroid
NEAT1	rs10896015	G_A	1.30×10^{-5}	0.34	Adipose - Subcutaneous
NEAT1	rs10896015	G_A	2.50×10^{-7}	0.34	Adipose - Visceral (Omentum)
NEAT1	rs10896015	G_A	5.30×10^{-9}	0.27	Artery - Aorta
NEAT1	rs10896015	G_A	4.20×10^{-7}	0.19	Artery - Tibial
NEAT1	rs10896015	G_A	5.00×10^{-6}	0.16	Cells - Transformed fibroblasts
NEAT1	rs10896015	G_A	3.60×10^{-8}	0.4	Colon - Transverse
NEAT1	rs10896015	G_A	7.50×10^{-10}	0.32	Esophagus - Mucosa
NEAT1	rs10896015	G_A	7.60×10^{-10}	0.3	Esophagus - Muscularis
NEAT1	rs10896015	G_A	4.40×10^{-5}	0.2	Heart - Atrial Appendage
NEAT1	rs10896015	G_A	4.40×10^{-5}	0.21	Heart - Left Ventricle
NEAT1	rs10896015	G_A	1.00×10^{-8}	0.25	Lung
NEAT1	rs10896015	G_A	6.20×10^{-10}	0.23	Muscle - Skeletal
NEAT1	rs10896015	G_A	1.20×10^{-13}	0.36	Nerve - Tibial
NEAT1	rs10896015	G_A	2.50×10^{-5}	0.28	Pituitary
NEAT1	rs10896015	G_A	2.30×10^{-6}	0.25	Skin - Not Sun Exposed (Suprapubic)
NEAT1	rs10896015	G_A	4.20×10^{-7}	0.22	Skin - Sun Exposed (Lower leg)
NEAT1	rs10896015	G_A	3.90×10^{-7}	0.34	Stomach
NEAT1	rs10896015	G_A	4.00×10^{-7}	0.21	Thyroid
PCNXL3	rs10896015	G_A	1.20×10^{-4}	-0.11	Nerve - Tibial
RNASEH2C	rs10896015	G_A	2.00×10^{-8}	-0.18	Muscle - Skeletal
RP11-770G2.4	rs10896015	G_A	2.60×10^{-5}	0.3	Testis
SSSCA1-AS1	rs10896015	G_A	2.80×10^{-5}	0.28	Adipose - Subcutaneous
SSSCA1-AS1	rs10896015	G_A	1.50×10^{-6}	0.24	Artery - Tibial
SSSCA1-AS1	rs10896015	G_A	2.70×10^{-7}	0.7	Cells - EBV-transformed lymphocytes
SSSCA1-AS1	rs10896015	G_A	2.10×10^{-5}	0.32	Colon - Sigmoid
SSSCA1-AS1	rs10896015	G_A	4.00×10^{-6}	0.23	Muscle - Skeletal
SSSCA1-AS1	rs10896015	G_A	7.10×10^{-5}	0.25	Skin - Sun Exposed (Lower leg)
SSSCA1-AS1	rs10896015	G_A	2.10×10^{-6}	0.28	Thyroid

NES: normalized effect size.
